# Supplementary material for: Predicting synthetic mRNA stability using massively parallel kinetic measurements, biophysical modeling, and machine learning
Source: Nat Commun. 2024 Nov 6;15:9601. doi: 10.1038/s41467-024-54059-7 (PMC11541907; doi:10.1038/s41467-024-54059-7)
Supplement: Supplementary file 1 — Supplementary Information [file 41467_2024_54059_MOESM1_ESM.pdf]

SUPPLEMENTARY INFORMATION

Predicting synthetic mRNA stability using massively parallel kinetic measurements, biophysical modeling, and machine learning

Daniel P. Cetnar[1], Ayaan Hossain[2], Grace E. Vezeau[3], and Howard M. Salis[1,3,4*]

[1]Department of Chemical Engineering, [2]Department of Bioinformatics and Genomics, [3]Department of Biological Engineering, [4]Department of Biomedical Engineering. The Pennsylvania State University, University Park, PA, 16802.

* To whom correspondence should be addressed: salis@psu.edu

Supplementary Table 1: Total reads, mapped barcode reads, and statistics for each DNA and RNA sample collected at timepoints T0 to T16.

Sample	Total Reads	Barcode Reads	Barcode Concordance (%)	Spike-In Reads	Spike-In Concordance (%)	Total Concordance (%)
T0	387,660,194	342,662,787	88.39	12,660,270	3.27	91.66
T2	369,864,886	298,093,097	80.60	44,437,786	12.01	92.61
T4	380,625,442	308,677,107	81.10	44,280,216	11.63	92.73
T8	206,006,733	157,050,048	76.24	33,660,927	16.34	92.58
T16	444,108,862	290,332,839	65.37	126,183,744	28.41	93.79
DNA	324,093,068	301,007,928	92.88	0	0.00	92.88

Supplementary Table 2: Hyperparameters used to train all LightGBM models.

Number of Leaves	100
Minimum Datapoints per Leaf	50 [prevents over-fitting]
Maximum Tree Depth	5 layers [prevents over-fitting]
Maximum Bins per Numerical Feature	1000
Number of Estimators	119
Learning Rate	0.10
Bagging Fraction	0.50 [prevents over-fitting]
Bagging Frequency	5 [prevents over-fitting]
Feature Fraction	0.25
Importance Type	Gain
Loss Metric	L2 Norm

Supplementary Figure 1: Single Design Factor Analysis of RppH Binding Sites. Measured mRNA decay rates across each grouping of characterized mRNAs with different 4-nt RppH binding site sequences. Green dots are mean mRNA decay rates within each category. Upper and lower quartiles are the tops and bottoms of black bars. The differences in the quartiles across categories are larger than the differences in the means.

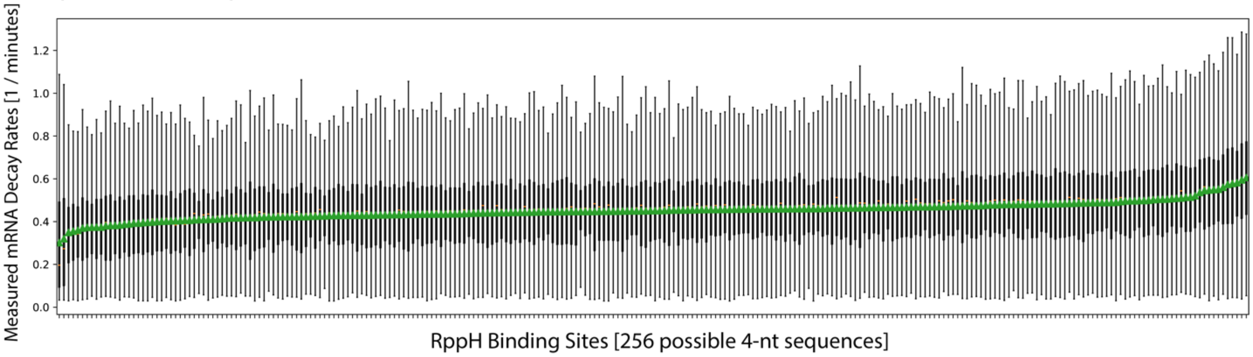

Supplementary Figure 2: Single Design Factor Analysis of mRNA Translation Rates. Measured mRNA decay rates across each grouping of characterized mRNAs with different predicted CDS translation initiation rates. Green dots are mean mRNA decay rates within each category. Upper and lower quartiles are the tops and bottoms of black bars. The differences in the quartiles across categories are larger than the differences in the means.

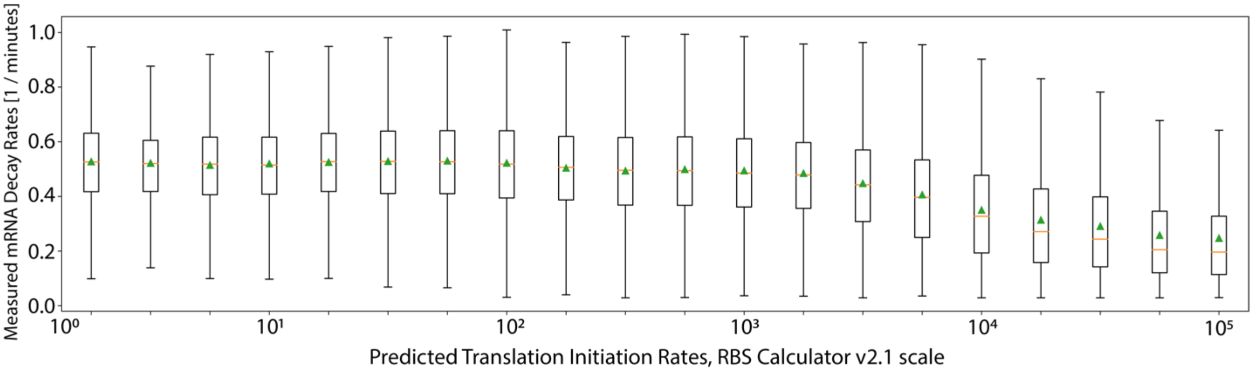

Supplementary Figure 3: Single Design Factor Analysis of Single-stranded RNA Lengths. Measured mRNA decay rates across each grouping of characterized mRNAs with different single-stranded RNA lengths inside the 5' UTR region. Upper and lower quartiles are the tops and bottoms of black bars. The differences in the quartiles across categories are much larger than the differences in the means.

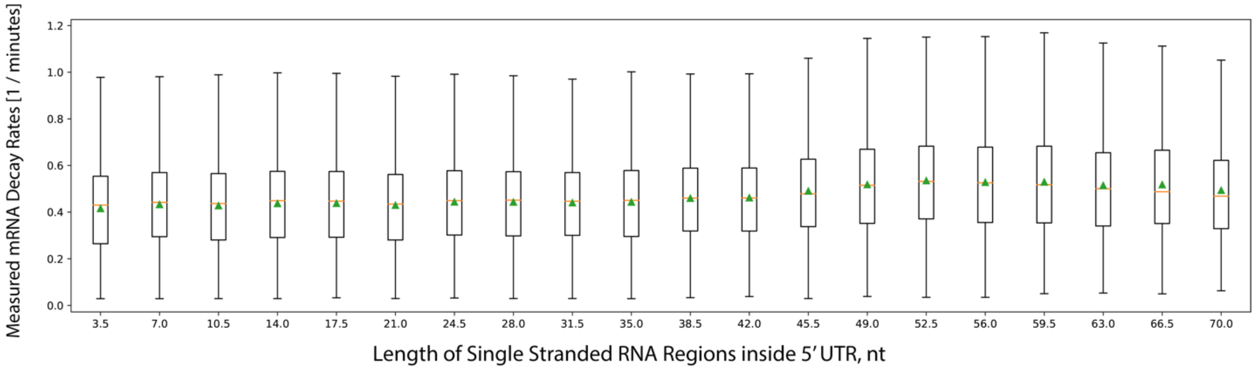

Supplementary Figure 4: Candidate mRNA structural features that were pruned from the list of isoform-specific features during development of the machine learning model.

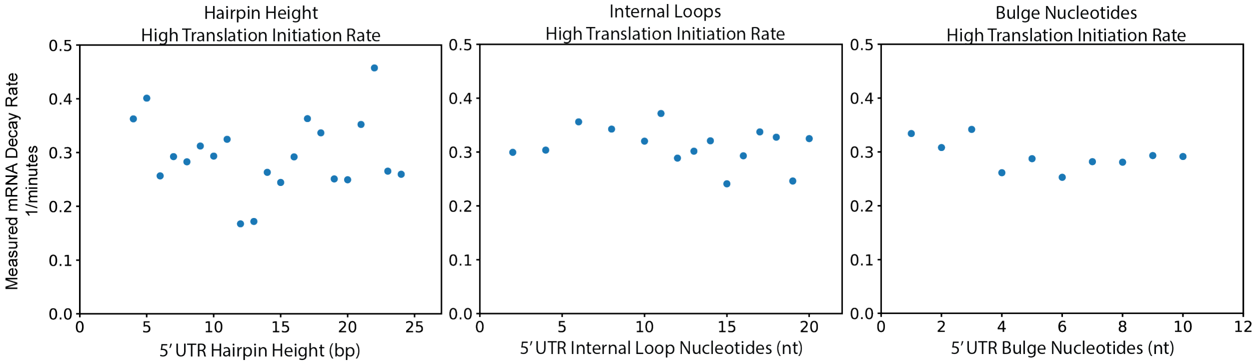

Supplementary Figure 5: The LightGBM-calculated importances of each isoform-specific feature in the finalized model when predicting the steady-state mRNA levels. The top 5 mRNA isoforms are numbered from 0 to 4.

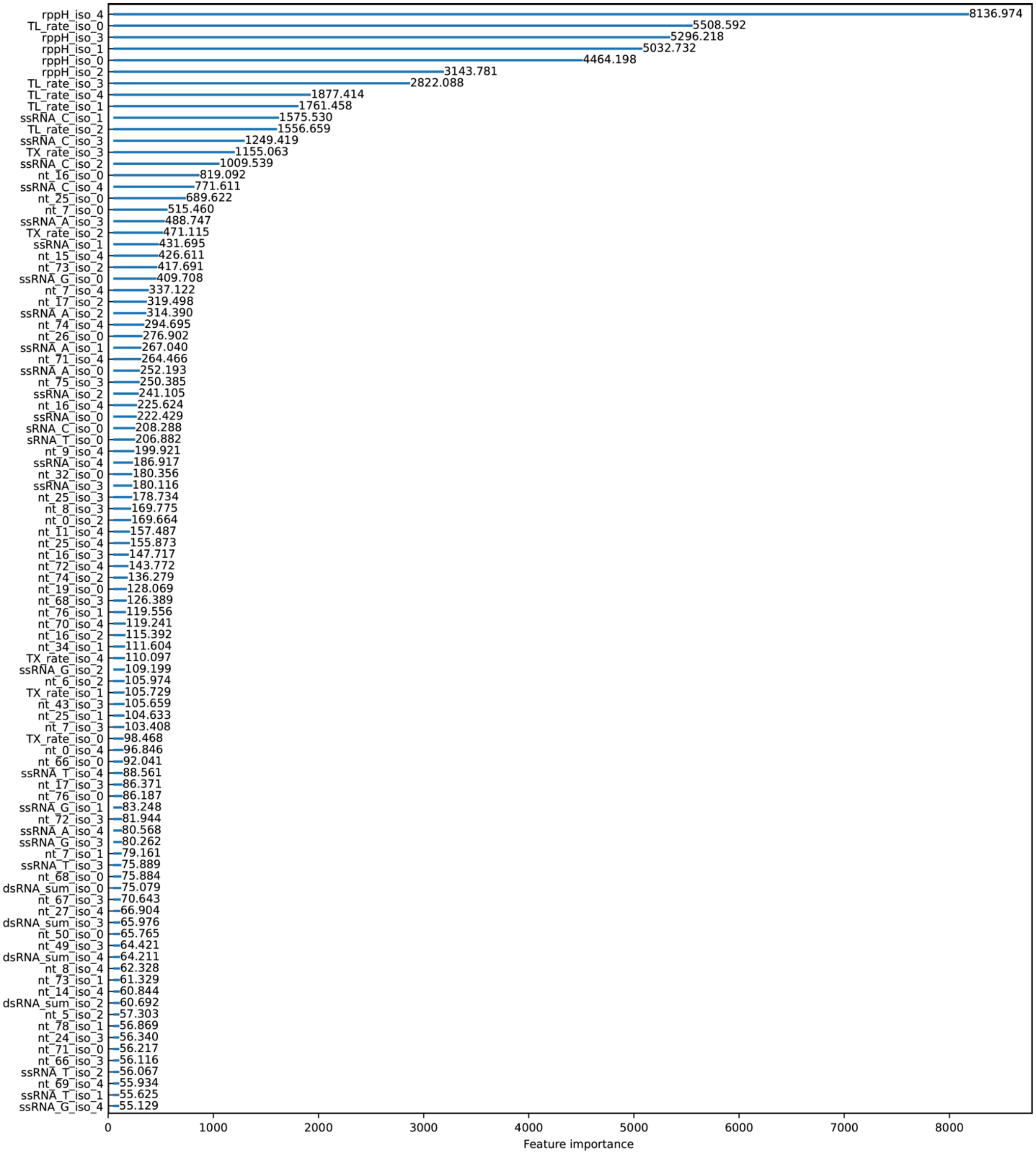

Supplementary Figure 6: The measured steady-state mRNA levels are compared to the model-predicted steady-state mRNA levels for characterized mRNAs in individual design groups.

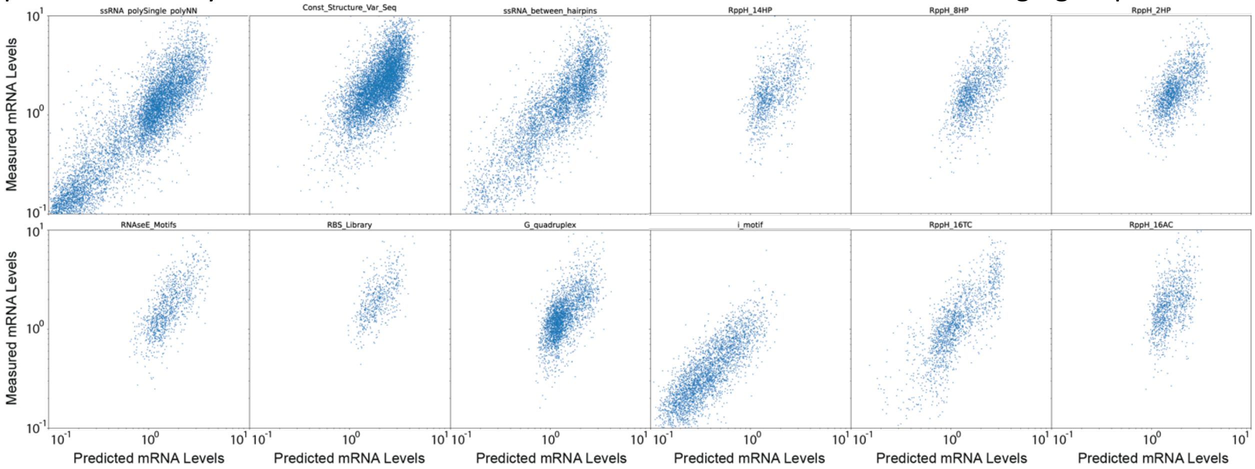

Supplementary Figure 7: Model-predicted steady-state mRNA levels when systematically varying the RppH binding site of a baseline mRNA with high stability.

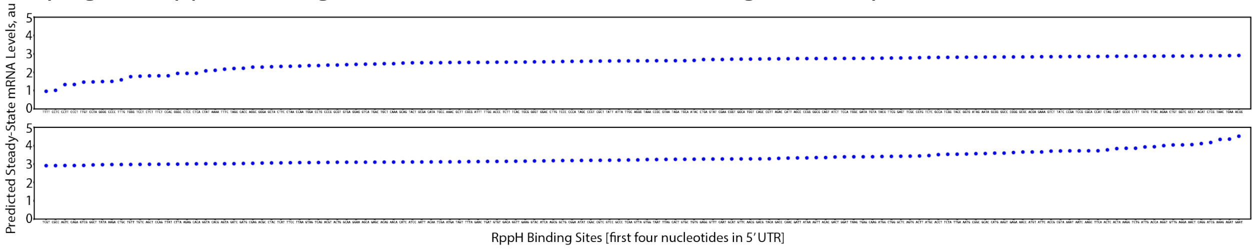

Supplementary Figure 8: The mRNAs' measured decay rates are compared to the inverse of the measured steady-state mRNA levels, showing a noisy non-linear relationship. Pearson R = 0.04.

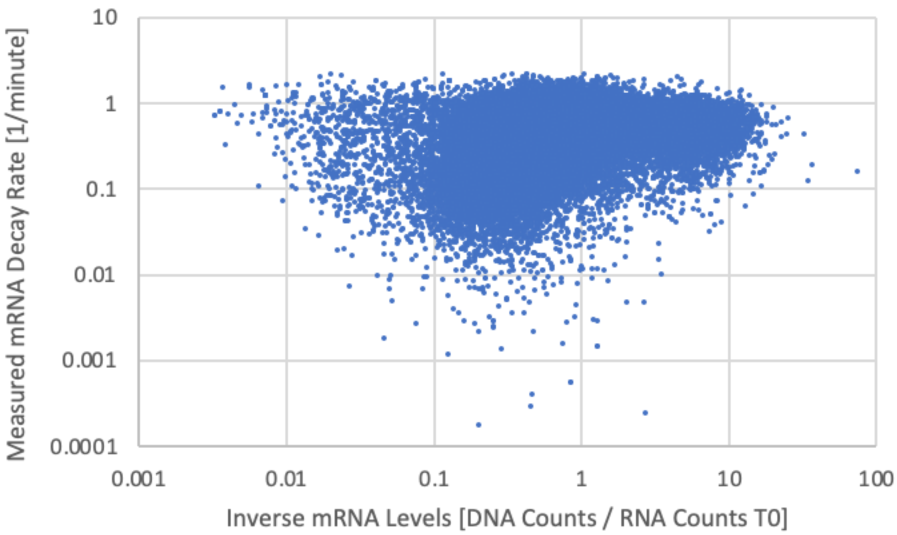

Supplementary Code 1: Python v3 code to determine mRNA decay rates from DNA-Seq read counts, RNA-Seq read counts, and Spike-in control RNA read counts, using SciPy and scikit-learn.

```
import numpy as np
import pandas as pd
from scipy.optimize import curve_fit
from sklearn.metrics import r2_score

def decay_fcn(x, k):
    return np.exp(-k * x)

def calculateDecayRates():

    filename = 'SupplementaryData1.xlsx'
    mydata = pd.read_excel(filename)

    DNA_T0 = mydata['DNA']
    RNA_T0 = mydata['RNA_T0']
    RNA_T2 = mydata['RNA_T2']
    RNA_T4 = mydata['RNA_T4']
    RNA_T8 = mydata['RNA_T8']
    RNA_T16 = mydata['RNA_T16']

    SpikeIn = [12660270, 44437786, 44280216, 33660927, 126183744]
    SpikeInRatios = [sp / SpikeIn[0] for sp in SpikeIn]
    timepoints = np.array([0.0, 2.0, 4.0, 8.0, 16.0], dtype=np.float64)

    MIN_T0_READS = 100.0
    MIN_T2_READS = 10.0
    MIN_T4_READS = 1.0
    MIN_T8_READS = 1.0
    MIN_T16_READS = 0.0

    analysis = []
    for n in range(len(DNA_T0)):

        if RNA_T0[n] < MIN_T0_READS or RNA_T2[n] < MIN_T2_READS or RNA_T4[n] < MIN_T4_READS or 
RNA_T8[n] < MIN_T8_READS or RNA_T16[n] < MIN_T16_READS:
            analysis.append({'k_fit' : 0.0, 'k_cond' : -1.0, 'k_r2_fit' : 0.0 })
        else:
            ydata1 = np.array([ RNA_T0[n]/RNA_T0[n]/SpikeInRatios[0],
                                RNA_T2[n]/RNA_T0[n]/SpikeInRatios[1],
                                RNA_T4[n]/RNA_T0[n]/SpikeInRatios[2],
                                RNA_T8[n]/RNA_T0[n]/SpikeInRatios[3],
                                RNA_T16[n]/RNA_T0[n]/SpikeInRatios[4]
                               ], dtype=np.float64)

            popt, pcov = curve_fit(decay_fcn, timepoints, ydata1)
            k = popt[0]
            cond = np.linalg.cond(pcov)

            y_pred = decay_fcn(timepoints, k)
            y_r2 = r2_score(ydata1, y_pred)

            print('k: {}. cond: {}. r^2: {}'.format(k,cond,y_r2))
            analysis.append({'k_fit' : k, 'k_cond' : cond, 'k_r2_fit' : y_r2 })

    df = pd.DataFrame(analysis)
    df.to_csv('output.csv')
```
