# Supplementary material for: Predicting synthetic mRNA stability using massively parallel kinetic measurements, biophysical modeling, and machine learning
Source: Nat Commun. 2024 Nov 6;15:9601. doi: 10.1038/s41467-024-54059-7 (PMC11541907; doi:10.1038/s41467-024-54059-7)
Supplement: Supplementary file 2 — Description of Additional Supplementary Files [file 41467_2024_54059_MOESM2_ESM.pdf]

Description of Additional Supplementary Information

Supplementary Data 1 contains sequence information and experimental measurements for all characterized genetic systems.

Supplementary Data 2 contains featurization, model calculations, and model predictions for all characterized genetic systems.
